# Structures and Applications of Thermoresponsive Hydrogels and Nanocomposite-Hydrogels Based on Copolymers with Poly (Ethylene Glycol) and Poly (Lactide-*Co*-Glycolide) Blocks

**DOI:** 10.3390/bioengineering6040107

**Published:** 2019-11-21

**Authors:** Tomoki Maeda

**Affiliations:** 1 Frontier Research Center for Applied Atomic Sciences, Ibaraki University, 162-1 Shirakata, Tokai, Ibaraki 319-1106, Japan; tomoki.maeda.polymer@vc.ibaraki.ac.jp; 2 Department of Mechanical Engineering, Keio University, 3-14-1, Hiyoshi, Kohoku-ku, Yokohama 223-8522, Japan

**Keywords:** PEG, PLGA, block copolymer, laponite, thermoresponsive hydrogel, nanocomposite, degradation, drug delivery, regeneration medicine, structural analysis

## Abstract

Thermoresponsive hydrogels showing biocompatibility and degradability have been under intense investigation for biomedical applications, especially hydrogels composed of hydrophilic poly(ethylene glycol) (PEG) and hydrophobic poly(lactic acid-*co*-glycolic acid) (PLGA) as first-line materials. Even though various aspects such as gelation behavior, degradation behavior, drug-release behavior, and composition effect have been studied for 20 years since the first report of these hydrogels, there are still many outputs on parameters affecting their gelation, structure, and application. In this review, the current trends of research on linear block copolymers composed of PEG and PLGA during the last 5 years (2014–2019) are summarized. In detail, this review stresses newly found parameters affecting thermoresponsive gelation, findings from structural analysis by simulation, small-angle neutron scattering (SANS), etc., progress in biomedical applications including drug delivery systems and regeneration medicine, and nanocomposites composed of block copolymers with PEG and PLGA and nanomaterials (laponite).

## 1. Introduction

Copolymers composed of hydrophilic poly(ethylene glycol) (PEG) and hydrophobic poly(lactic acid-*co*-glycolic acid) (PLGA) are widely studied for biomedical applications due to their thermoresponsive gelation, biocompatibility, and degradability. PLGA–PEG–PLGA triblock copolymer solution shows thermoresponsive gelation around physiological temperature (25–37 °C). Biocompatibility has also been proved, because both PEG and PLGA are approved by the US Food and Drug Administration (FDA). Furthermore, degradability is a characteristic of PLGA, a biodegradable polyester, and the degradation rate can be easily controlled by the LA/GA ratio (i.e., the molar ratio of lactide (LA) to glycolide (GA)). Controlled degradation leads to controlled drug release. Degradation cannot be achieved in other types of thermoresponsive hydrogels such as poly(N-isopropylacrylamide) (PNIPAAM) and PEG-poly(propylene glycol) (PPG), and the parameter to control the degradation of thermoresponsive hydrogels based on copolymers with PEG and PLGA blocks (i.e., the ratio to PEG to PLGA (PEG/PLGA ratio) and the LA/GA ratio) is more than that of PEG-polycaprolactone (PCL) block copolymers (i.e., the ratio to PEG to PCL (PEG/PCL ratio)).

Since the first reports of biodegradable thermoresponsive hydrogels composed of PEG and poly(L-lactic acid) (PLLA) and PEG–PLGA–PEG by Jeong et al. in 1997 and 1999, respectively [1,2], characteristics of aqueous copolymer solutions such as gelation temperature, degradation rate, and release rate were studied by changing the solute and chemical composition. Originally, the characteristics of aqueous solutions of PEG–PLGA–PEG triblock copolymers were reported by Jeong et al. [2,3,4]. In the reports, thermoresponsive gelation behavior, micellization behavior (e.g., critical micelle concentration (CMC) and micelle diameter), and degradation behavior were investigated by changing the solution concentration, the molecular weight of PEG and PLGA blocks, and the composition of PLGA. In detail, the CMC was confirmed to be ~0.01 wt%. As for the degradation process, the process was proposed as follows. More hydrophilic PEG-rich blocks derived by the degradation of PEG–PLGA–PEG are preferentially diffused out of the gel during degradation. In addition, the time scale of degradation was confirmed to be over 30 days.

After the series of studies on PEG–PLGA–PEG, PLGA–PEG–PLGA triblock copolymers have been widely studied by Lee et al. [5,6] and Zentner et al. [7]. Basically, the same as PEG–PLGA–PEG, the characteristics of thermoresponsive hydrogels (i.e., gelation behavior, micellization, degradation behavior) were studied. In addition to the basic properties, the gelation process was proposed and discussed using PLGA–PEG–PLGA [5,6]. A brief explanation of the gelation process is as follows. In the aqueous solution of block copolymers, micelles are formed due to the hydrophobic effect of PLGA blocks. When the solution temperature is increased, the hydrophobic interactions between micelles become stronger, and networks or aggregates of micelles are formed. At the same time, utilization as a delivery system for proteins and water-insoluble drugs was also proposed and discussed [8,9,10]. Moreover, the compositional effects (i.e., the end-group effect [11,12] and the effect derived from minor sequential differences in PLGA blocks [13]) and the blend system of PLGA–PEG–PLGA with different block ratios [14,15]) were further investigated.

According to the previous studies during 1997–2012 mentioned above, we can learn details from comprehensive reviews reported by Alexander et al., Zhang et al., and Wang et al. [16,17,18]. Therefore, in this review, current trends of research on block copolymers composed of PEG and PLGA during the last 5 years (2014–2019) including structural analysis and applicational study are summarized. First, new parameters affecting thermoresponsive gelation are discussed. Then, the findings from new analytical methods and simulation are discussed. Moreover, research on biomedical applications including drug delivery systems and regeneration medicine are discussed. Finally, nanocomposites composed of block copolymers composed of PEG and PLGA and nanomaterials (laponite) are discussed.

## 2. Parameters Affecting Thermoresponsive Gelation

Parameters affecting thermoresponsive gelation have been widely studied [5], however, there are still some newly found parameters. According to comprehensive studies, the solution concentration, the molecular weights of PEG and PLGA blocks, and the composition of PLGA are known to be basic parameters affecting thermoresponsive gelation. In this section, these new parameters found in 2014–2019 are summarized.

Table 1 shows a summary of studies from 2014–2019 on the parameters affecting thermoresponsive gelation. As for the additives, salt often plays an important role in aqueous solutions. Li et al. first reported the gel-to-sol-to-(re-entrant gel) transitions of PEG–PLGA aqueous solution in the presence of salts. The transition temperatures varied with the salt concentration and depended on the type of ion, with the trend obeying the Hofmeister series. They also anticipated that virgin gel was formed by the jamming of close-packed micelles, while re-entrant gel was formed by the percolated micelle network [19].

As for the composition of block copolymers, although the molecular weights of PEG and PLGA blocks were known to be a key factor, the effect of the molecular weight distribution (MWD) on gelation behavior was unclear before 2014. Chen et al. first reported the effects of MWD on thermoresponsive gelation and related behaviors of PLGA–PEG–PLGA aqueous solutions. They prepared a series of PLGA–PEG–PLGAs with different MWDs under similar weight-average molecular weight (Mw) or number-average molecular weight (Mn) via step precipitation and mixing of the synthesized copolymers with a given PEG length and varied PLGA lengths. They revealed that increased MWD enhanced the solubility of PLGA–PEG–PLGA in water and increased the critical micelle concentration (CMC), and the critical gelation concentration (CGC) and sol-gel transition temperature (T_gel_) increased with MWD under both a given Mw and a given Mn [20]. Then, Chen et al. also reported the effects of MWD of hydrophilic PEG blocks on the corresponding gelation behavior of PLGA–PEG–PLGA aqueous solutions. They prepared a series of PLGA–PEG–PLGAs with similar PLGA blocks but varied length and MWD of the PEG block. They revealed that the gel-to-sol or sol-to-gel transition occurred only with appropriate Mw and MWD, and that the wider MWD of the PEG block sometimes led to the coexistence of the sol-to-gel transition upon cooling and upon heating [21]. As mentioned above, the molecular weights of PEG and PLGA blocks were known to be a key factor, however, the effect of PEG and PLGA molecular weights was not comprehensively investigated. Recently, Steinman et al. first reported the effect of the PLGA/PEG ratio and PEG molecular weight on T_gel_ of PLGA–PEG–PLGA aqueous solution (20 wtr%). They revealed a linear relation between T_gel_ and the PLGA/PEG ratio and variation in Tgel dependence on the PLGA/PEG ratio based on PEG molecular weight [22].

Especially for PEG–PLGA–PEG triblock copolymer, with hydrophobic PLGA middle block and two hydrophilic PEG end blocks, the structure of the coupling agents in the middle of PEG–PLGA–PEG chains introduced through the copolymer’s synthesis from PEG–PLGA diblock copolymers was found to be important. Luan et al. reported the positional isomeric effects of coupling agents in the middle of PEG–PLGA–PEG on the macroscopic conformation and properties of thermoresponsive block copolymers. They prepared PEG–PLGA–PEG with positional isomers in the middle of polymer chains by linking PEG–PLGA with different difunctional positional isomers as the coupling agents (*o*-PC, *m*-PC, and *p*-PC; PC, phthaloyl dichloride). They revealed that PEG–PLGA–PEG with *o*-PC, *m*-PC, and *p*-PC exhibited lower T_gel_ and larger moduli in gel state due to the phenyl rigidity of PC as compared to PEG–PLGA–PEG with hexamethylene diisocyanate (HMDI), a conventional coupling agent. They also revealed that PLGA–PEG–PLGA coupled with *o*-PC had a smaller coil size and exhibited lower T_gel_ and higher modulus as compared to PLGA–PEG–PLGA coupled with *m*-PC and *p*-PC [23]. Thus, comprehensive studies on the parameters affecting thermoresponsive gelation are still going on for further fine regulation of hydrogel properties.

## 3. Structural Analysis of Hydrogels Composed of Block Copolymers with PEG and PLGA Blocks

For structural analysis of thermoresponsive hydrogels composed of block copolymers with PEG and PLGA blocks, the dispersion state of micelles (i.e., the diameter of micelles and size of aggregated micelles) was mainly analyzed by dynamic light scattering (DLS) and transmission electron microscopy (TEM). In previous studies, DLS measurements were carried out at relatively lower concentration below the critical gelation concentration (CGC), or at relatively lower temperature below the gelation temperature (T_gel_). This is because, in general, samples for DLS analysis should be liquid and transparent. In other words, DLS is unavailable for concentrated or turbid systems such as opaque hydrogels. As for TEM analysis, samples at relatively lower concentrations below CGC were used and observations were made in the dry state. Due to the limited approach and limited state of samples for structural analysis, we could not share a clear image of the structural change induced through thermoresponsive gelation. 

Owing to the development of analytical and simulation methods, there are still some new findings on the structure and dynamics of thermoresponsive hydrogels. As far as analytical methods, small-angle neutron scattering (SANS), 3D DLS, and fluorescence resonance energy transfer (FRET) techniques were introduced. As far as simulation methods, dissipative particle dynamics (DPD) simulation and Monte Carlo simulation were introduced. In this section, research on the structural analysis of hydrogels composed of block copolymers with PEG and PLGA blocks during 2014–2019 is summarized.

**Figure 1:**
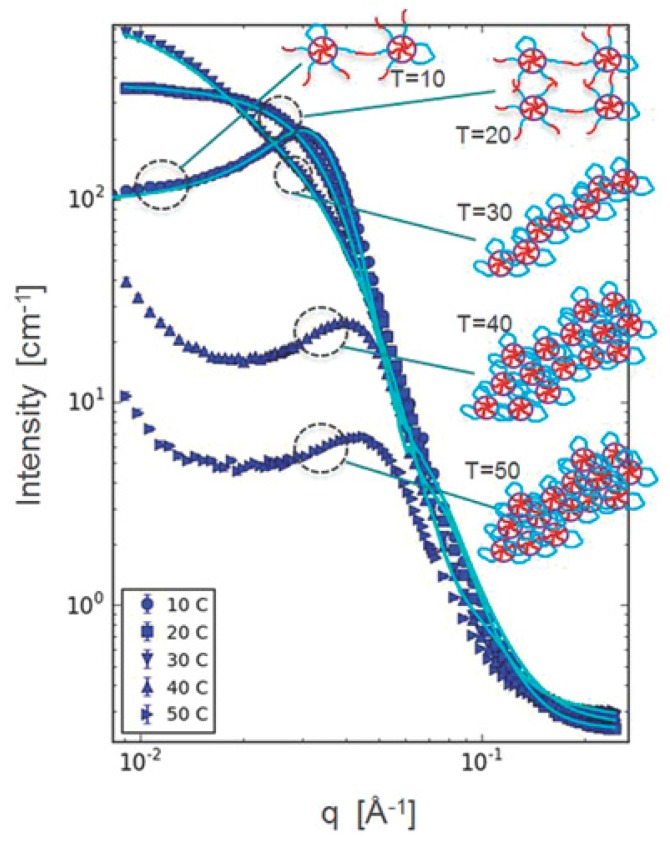
Structural analysis of PLGA–PEG–PLGA solution (20 wt%) by SANS. Reprinted from [24] Micromolecular Bioscience, 16, Neda Khameh Khorshid et al., Novel structural changes during temperature induced self-assembling and gelation of PLGA-PEG-PLGA triblock copolymer in aqueous solutions, 1838–1852, Copyright (2016), with permission from Wiley.

Table 2 shows a summary of the research on structural analysis of thermoresponsive hydrogels composed of block copolymers with PEG and PLGA blocks during 2014–2019. SANS is a powerful method for structural analysis of concentrated hydrogels due to the high transmittance of neutrons through the samples and high contrast between the copolymer and water by using D_2_O instead of H_2_O. As for the results revealed by SANS, Khorshid et al. reported the structural change of micelles and the gel network in response to increased temperature using concentrated solution (Figure 1). They revealed by SANS that the correlation peak suggesting ordered structure with a characteristic intermicellar distance disappeared and a less organized structure appeared in the gel region, and that a cylindrical structure was established at higher temperature and disorderly packing of cylinders occurred at still higher temperature [24]. For diluted samples, they also revealed differences in the micellar morphology: PLGA–PEG–PLGA with short PEG block (Mn ~1000 g/mol) formed asymmetric (ellipsoid) micelles, whereas PLGA–PEG–PLGA with long PEG block (Mn ~1500 g/mol) formed spherical micelles.

Along with SANS, 3D-DLS is also a powerful method to analyze concentrated solutions or turbid systems with multiple scattering as compared to conventional DLS, which can be applied to samples with single scattering. In reports by Cui et al. [25], the thermoresponsive gelation behavior of PEG–PLGA solution was characterized by 3D DLS. They confirmed the existence of larger and highly irregular clusters for the concentrated system (25 wt%) as compared to the low-concentration solution (1 wt%) In their report, a connected micellar structure in thermoresponsive hydrogels was also detected by fluorescence resonance energy transfer (FRET). FRET is a method to detect nanoscale distance using a FRET pair (a donor and an acceptor). By mixing the solution containing micelles with a donor and an acceptor, the donor and acceptor were isolated by micelles. When the connected micellar structures were created, the distance of the donor and acceptor decreased due to diffusion through the connected structure. Thus, the dynamics during thermoresponsive gelation and the local structure of the micellar network were accurately investigated by the introduction of new analytical methods.

**Figure 2:**
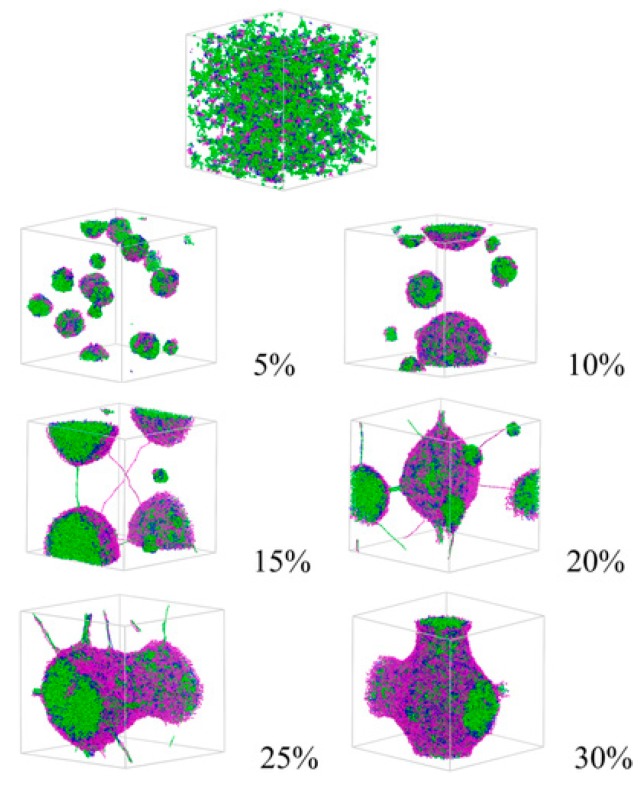
Morphology of PLGA–PEG–PLGA in water at different concentrations revealed by DPD simulation. Reprinted from Journal of Applied Polymer Science, 132, Yang Cao et al., In Vitro evaluation and dissipative particle dynamics simulation of PLGA–PEG–PLGA, 41280, Copyright (2014), with permission from Wiley.

**Figure 3:**
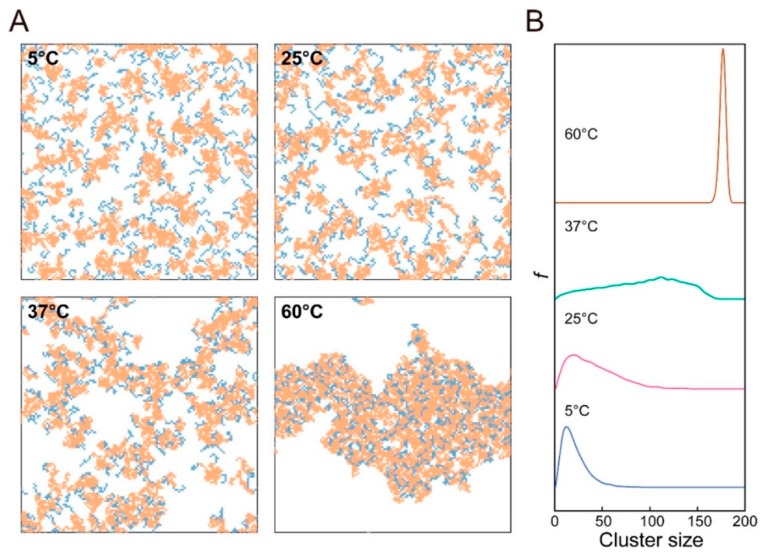
Mesoscopic structure of PEG–PLGA in water at different temperatures revealed by Monte Carlo simulation: (**A**) typical snapshots of the systems and (**B**) corresponding cluster size distribution. Reprinted from [25] Macromolecules, 51, Shuquan Cui et al., Semi-bald micelles and corresponding percolated micelle networks of thermogels, 6405–6420, Copyright (2018) American Chemical Society.

Development of simulations supported by development of high-performance computers enabled us to explore the morphology of polymers at a molecular level. As for the results revealed by simulation, an exploration of the morphological transition of PLGA–PEG–PLGA by DPD simulation was carried out by Cao et al. [26]. DPD simulation is a mesoscopic simulation method for complex fluid and has been used since 1992. It was revealed that small spherical micelles were observed at concentrations of 5 wt% and 10 wt%, and these micelles united into a larger one, with the concentration increasing up to 20%, and a columnar structure was formed at concentrations of 25 wt% and 30 wt% (Figure 2). Monte Carlo simulation was also carried out by Cui et al. [25] in order to reveal the mechanism behind reversed thermoresponsive gelation with sol-gel transition upon heating. In their system, with a new type of micelle, the semi-bald micelle, as precursor for thermoresponsive gelation, they demonstrated that the structure of thermoresponsive hydrogels was a percolated micelle network with hydrophobic channels that evolved from semi-bald micelles (Figure 3). Thus, they first demonstrated the mesoscopic structure of amphiphilic copolymer chains in water during thermoresponsive gelation. To summarize, computer simulation helps us understand the results from experiments and obtain clear images of the hierarchical structure.

## 4. Applications of Thermoresponsive Hydrogels Using Block Copolymers with PEG and PLGA Blocks

Since the invention of thermoresponsive hydrogels, research on various biomedical applications has been consistently carried out. It seems that research on the applications shifted to a more practical phase. In this section, research on more practical biomedical devices using block copolymers with PEG and PLGA blocks during 2014–2019 is summarized.

Table 3 shows a summary of the research on more practical biomedical devices using block copolymers with PEG and PLGA blocks during 2014–2019. Conventionally, the main applications of thermoresponsive hydrogels have been drug delivery systems. From the background, the major research on the application of thermoresponsive hydrogels is still on drug delivery systems. In order to obtain controlled release of water-soluble drugs, various strategies using polymer–drug conjugates, nanoparticles, microparticles, and vesicles/emulsomes have been suggested. In addition, regarding the drugs used in drug delivery systems, not only chemical compounds but also peptide/protein drugs have been widely studied. As another application of hydrogels, utilization as cell scaffold is major. Thus, utilization of thermoresponsive hydrogels for tissue regeneration has also been reported. Furthermore, based on thermoresponsive gelation properties, some unique applications such as submucosal cushion for endoscopic submucosal dissection (ESD), a device to prevent postoperative adhesions, and a dressing for cutaneous wound healing have been studied.

The basic drug delivery system prepared just by mixing drugs into thermoresponsive hydrogels was widely studied for the treatment of cancer/tumors, ophthalmic disease, viral diseases, helminthic diseases, detoxification of opioids and alcohol, and postoperative pain relief. For the treatment of cancer/tumors, doxorubicin (DOX) is a well-studied drug released from thermoresponsive hydrogels among anticancer drugs. The maximum tolerated dose (MTD) of DOX with PLGA–PEG–PLGA hydrogels was investigated by Yang et al. [27]. As for anticancer drugs other than DOX, the delivery system of irinotecan (IRN), a clinically used antitumor drug with moderate solubility, was investigated by Ci et al. [28]. It was revealed that excellent in vivo antitumor efficacy was observed in the group that received IRN-loaded thermoresponsive hydrogel, while side effects such as blood toxicity and decreased body weight were very mild. For the treatment of ophthalmic disease, drug delivery systems composed of thermoresponsive hydrogels are also widely studied. This is due to the restricted permeability and fast clearance of drugs induced by the complex anatomy and physiology of the eye [29]. For example, a delivery system for dexamethasone (DEX), a hydrophobic glucocorticoid, was investigated for the treatment of posterior segment diseases by Zhang et al. [30]; a delivery system for cyclosporine A (CsA), an effective immunosuppressive agent, was investigated for the inhibition of postoperative scarring after filtration surgery for glaucoma by Sun et al. [31]; and a co-delivery system for metformin (MET), a potential agent for inhibiting neovascularization, and levofloxacin (LFH), a hydrophobic antibiotic for treating ocular infections and inflammatory responses, was investigated for the treatment of corneal neovascularization (CNV) by Liu et al. [32]. In addition, the excellent biocompatibility of PLGA–PEG–PLGA hydrogels and their capability as an ophthalmic drug delivery system were confirmed by Chan et al. [29]. Besides the treatment of cancer/tumors and ophthalmic disease, a delivery system for antivirals (cidofovir (CDV) and ganciclovir (GCV)) via the intratympanic route was investigated by Sidell et al. [33]; a delivery system for an effective agent against helminths (albendazole sulfoxide (ABZSO)) was investigated by Feng et al. [34]; a delivery system for an opioid antagonist (naltrexone hydrochloride) used in the maintenance phase of detoxification of opioids and alcohol in addicted patients was investigated by Mohajeri et al. [35]; and a delivery system for a local anesthetic (ropivacaine hydrochloride (RP)) for postoperative pain relief was investigated by Fu et al. [36].

**Figure 4:**
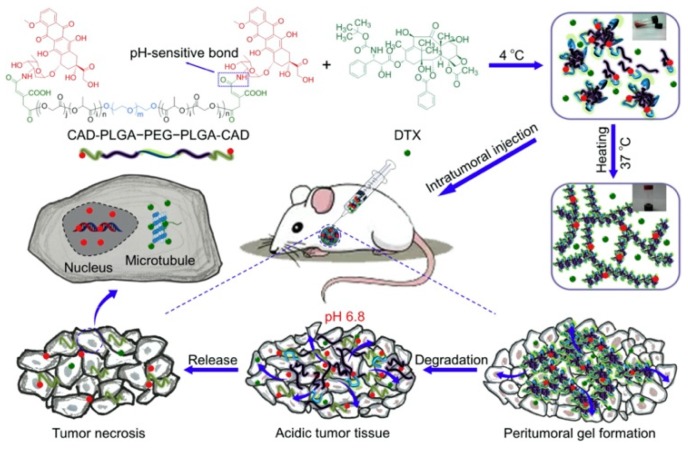
Schematic image of thermoresponsive hydrogels with PLGA–PEG–PLGA–drug conjugate. Reprinted from [37] Acta Biomaterialia, 77, Yanbo Zhang et al., Tumor microenvironment-labile polymer–doxorubicin conjugate thermogel combined with docetaxel for in situ synergistic chemotherapy of hepatoma, 63–73, Copyright (2018), with permission from Elsevier.

**Figure 5:**
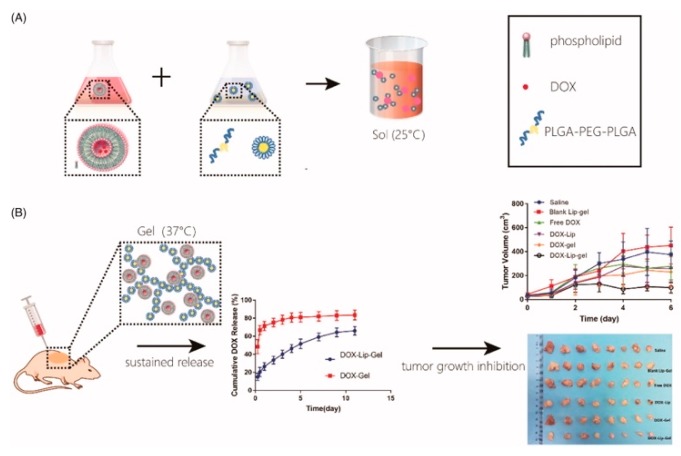
Schematic image of thermoresponsive hydrogels with vesicles/emulsomes: (**A**) a solution preparation method and (**B**) sustained release and tumor growth inhibition achieved by thermoresponsive hydrogels with vesicles/emulsomes. Reprinted from [38] ARTIFICIAL CELLS, NANOMEDICINE, AND BIOTECHNOLOGY, 47, Dinglingge Cao et al., Liposomal doxorubicin-loaded PLGA-PEG-PLGA based thermogel for sustained local drug delivery for the treatment of breast cancer, 181–191, Copyright (2019).

In order to obtain a drug delivery system with controlled release, the synthesis of PLGA–PEG–PLGA and drug conjugate and the fabrication of thermoresponsive hydrogels with nanoparticles, microparticles, or vesicles/emulsomes were examined. For example, since DOX is a small hydrophilic molecule, it would be released quickly due to the diffusion release mechanism. One method to control the diffusion of water-soluble DOX, DOX-conjugated PLGA–PEG–PLGA, was proposed by Zhang et al. (Figure 4) [37]. They also revealed obviously high antitumor efficacy and reliable security of thermoresponsive hydrogels composed of DOX-conjugated PLGA–PEG–PLGA (cis-aconitic anhydride-functionalized DOX (CAD)–PLGA–PEG–PLGA–CAD) showing acid sensitivity, and docetaxel (DTX), a microtubule-interfering agent. A different method to control the diffusion of water-soluble DOX is to incorporate water-soluble DOX into vesicles/emulsomes. For example, controlled release of DOX was achieved with hydrogels containing liposomal DOX by Cao et al. (Figure 5) [38]. They prepared liposomal DOX (DOX-lip) with a particle size of 74.6 nm and entrapment efficiency of 86% via the traditional film dispersion method and loaded the DOX-lip into PLGA–PEG–PLGA hydrogels. Sustained release of DOX up to 11 days was confirmed, without significant burst release and better antitumor efficacy and fewer side effects as compared to DOX-loaded hydrogels. The same method was applied to controlled release of another anticancer drug [39], a drug for the treatment of Parkinson syndrome and cocaine dependence [40], and an antiepileptic drug [41]. In detail, for a delivery system for cytarabine, an anticancer drug, investigated by Liu et al. [39], vesicles of ion-pair amphiphilic molecules were fabricated using cytarabine hydrochloride and sodium bis(2-ethylhexyl) sulfosuccinate (AOT), an anionic surfactant. Then, the prepared vesicles were incorporated into PLGA–PEG–PLGA hydrogels. For a delivery system for amantadine (AT), a drug for the treatment of Parkinson syndrome and cocaine dependence, investigated by Yang et al. [40], vesicles of ion-pair amphiphilic molecules using AT and oleic acid (OA) were fabricated, and the prepared AT–OA vesicles were incorporated into PLGA–PEG–PLGA hydrogels. For a delivery system for oxcarbazepine (OX), an antiepileptic drug, reported by Zaafarany et al. [41], OX-loaded emulsomes using phospholipids (PC), triolein (TO), and Tween 80 were prepared, then added to PLGA–PEG–PLGA hydrogels. The concept was also applied to the release of drugs with low bioavailability and multiple drugs. For example, for drugs with low bioavailability, the incorporation of brimonidine (Bri), a drug with ocular hypotensive effect and low bioavailability, onto layered double hydroxide (LDH) nanoparticles was investigated by Sun et al. [42]. They first prepared Bri–LDH nanoparticles using a modified hydrothermal process, then dispersed the nanoparticles into PLGA–PEG–PLGA hydrogel. As for multiple drugs, multi-delivery of ophthalmic aquatic agents at programmed rates and times was achieved by incorporating the drugs into microparticles and thermoresponsive hydrogels separately [43]. In the study, two drugs were loaded into microparticles, and the drug-loaded microparticles were encapsulated into thermoresponsive hydrogels containing another drug.

As for the drugs for drug delivery systems, peptide/protein drugs and genes should be considered because their use as therapeutic agents has increased considerably in recent years. As examples of using peptide/protein drugs, a delivery system for liraglutide (Lira), a fatty acid-modified antidiabetic polypeptide, for the treatment of diabetes was investigated by Chen et al. [44], and controlled release of growth hormone–releasing peptide 6 (GHRP-6) for the growth of rex rabbits was investigated by Guan et al. [45]. In order to prolong the bioactivity of peptide/protein drugs, an incorporation into microparticles and a complex formation were performed. As for the method of incorporating drugs into microparticles, Wang et al. proposed PLGA microsphere-loaded thermoresponsive hydrogels [46]. They prepared exenatide (EXT)-loaded hydrogels, encapsulated these hydrogels into PLGA microspheres, and further encapsulated the microspheres with EX-loaded hydrogels into blank hydrogel. As for the method of complex formation, a delivery system for human calcitonin (hCT), a peptide drug, was investigated by Shang et al. [47]. They prepared hCT–cucurbit 7 (CB7) complex (cucurbit 7 is an amphiphilic small molecule) to inhibit the fibrillation of highly amyloidogenic hCT by CB7, then incorporated the hCT-CB7 complex into PLGA–PEG–PLGA hydrogel. As another example, a delivery system for salmon calcitonin (sCT), a bioactive peptide to regulate serum calcium concentration by a therapeutic effect to improve bone mass and relieve osteoporotic bone pain, for long-term anti-osteopenia treatment was investigated by Liu et al. [48]. They prepared a complex of sCT and oxidized calcium alginate (OCA), a negatively charged polyelectrolyte, to stabilize sCT and control its affinity, then incorporated the sCT-OCA complex into PLGA–PEG–PLGA hydrogels. As an example using genes, an attempt to deliver a complex of PLK1shRNA and polylysine-modified polyethylenimine (PEI-Lys) (PLK1shRNA/PEI-Lys) was reported by Ma et al. [49]. They revealed that hydrogel containing PLK1shRNA/PEI-Lys and DOX exhibited significant synergistic effects in promoting apoptosis of osteosarcoma cells in vitro and superior antitumor efficacy in vivo. Thus, promising therapeutic effects achieved by the delivery of peptide/protein drugs or genes from thermoresponsive hydrogels composed of PEG and PLGA were revealed.

Along with drug delivery systems, tissue regeneration is also a target application of thermoresponsive hydrogels. In fact, there are many papers on bone tissue regeneration using thermoresponsive hydrogels. For example, bone regeneration was examined using a delivery system for simvastatin (SIM) from PLGA–PEG–PLGA hydrogels by Yan et al. [50]. They confirmed increased mineralization and osteogenic gene expression due to released SIM through an in vitro cell proliferation test. They also revealed that bone defects injected with SIM-loaded hydrogels showed enhancement of new bone formation. As an example using inorganic compounds, a delivery system for calcium cations by incorporating bioactive hydroxyapatite (HAp) in the form of micro- and nanoparticles (μ-HAp and n-HAp) for bone regeneration was reported by Chamradova et al. [51]. They confirmed controlled release of calcium cations from a system containing n-HAp without any initial burst release. As an example using protein, the treatment of critical-sized femoral defects was examined using a delivery system for bone morphogenetic protein (BMP)-2 from PEG–PLGA–2, 2′-bis(2-oxazolin) (Box)–PLGA–PEG hydrogels by Peng et al. [52]. They revealed that BMP-2-loaded hydrogel effectively promoted fracture healing, and that bone defect healing using the hydrogels with 20 μg/mL of BMP-2 was nearly equivalent to healing using autologous bone graft. A trial using stem cells was also carried out by Zhang et al. [53]. They reported the use of PLGA–PEG–PLGA hydrogel as a scaffold for bone marrow mesenchymal stem cells (BMMSCs) for repair of full-thickness articular cartilage defects. They confirmed first that the mechanical properties of PLGA–PEG–PLGA hydrogels were high enough to support the repair of cartilage, and biodegradability and biocompatibility were excellent. Then, they revealed that after implanting BMMSC-encapsulated PLGA–PEG–PLGA hydrogel into a full-thickness articular cartilage defect (diameter of 5.0 mm and depth of 4.0 mm), the regenerated cartilage integrated well with surrounding normal cartilage and subchondral bone at 12 weeks post-surgery. They also revealed upregulated expression of glycosaminoglycan and type II collagen, typical secretory products of chondrocytes in hyaline cartilage, in the repaired cartilage and comparable biomechanical properties with normal cartilage.

**Figure 6:**
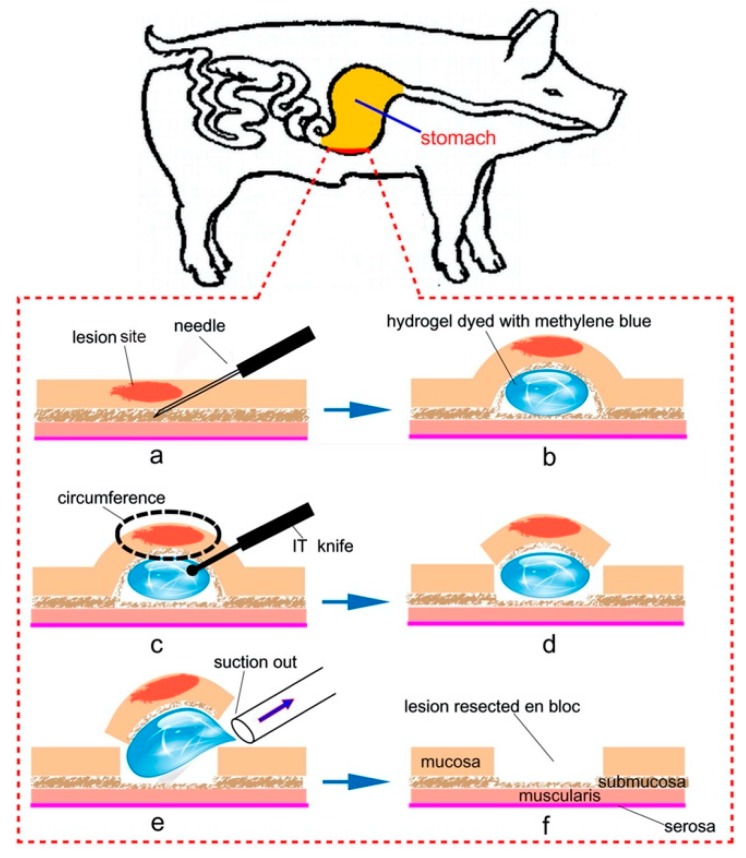
Schematic image of the application of thermoresponsive hydrogels as a submucosal cushion for endoscopic submucosal dissection (ESD): (**a**,**b**) injection of thermoresponsive hydrogels into the submucosal layer for mucosal elevation, (**c**,**d**) circumferential resection by cutting open via an insulation-tripped (IT) knife, and (**e**,**f**) suction of the gel under the mucosa and resection of lesion en bloc. Reprinted from [54] Acta Biomaterialia, 10, Lin Yu et al., Poly(lactic acid-*co*-glycolic acid)–poly(ethylene glycol)–poly(lactic acid-*co*-glycolic acid) thermogel as a novel submucosal cushion for endoscopic submucosal dissection, 1251–1258, Copyright (2013), with permission from Elsevier.

Some unique applications such as a submucosal cushion for endoscopic submucosal dissection (ESD), a device preventing postoperative adhesions, and a dressing for cutaneous wound healing have been suggested. Yu et al. introduced an injectable thermoresponsive hydrogel composed of PLGA–PEG–PLGA as a novel submucosal substance in ESD (Figure 6) [54]. They performed submucosal injection of thermoresponsive hydrogel for the formation of a submucosal fluid cushion (SFC) in resected porcine stomachs and living minipigs. Accurate en bloc resection was achieved as a result of high mucosal elevation, with a clear margin maintained for a long duration. Cao et al. also evaluated the efficacy of PLGA–PEG–PLGA hydrogels as a colonic submucosal agent [55]. They injected concentrated PLGA–PEG–PLGA aqueous solution into the colonic submucosa of living minipigs and confirmed adequate mucosal elevation lasting for a longer time than that created by using glycerol fructose, a highly viscous agent previously considered as a fluid for mucosal elevation. As for a biomedical device for postoperative care, Yu et al. reported on comparative studies of thermoresponsive hydrogels in preventing postoperative adhesions using three polyester–PEG–polyester triblock copolymers: PLGA–PEG–PLGA, PCGA–PEG–PCGA, and PCL–PEG–PCL [56]. They revealed that the PLGA–PEG–PLGA hydrogel (25 wt%) was most effective in reducing the formation of intraperitoneal adhesion as compared to PCGA–PEG–PCGA (25 wt%) and PCL–PEG–PCL (25 wt%) hydrogels. They also revealed that the viscoelasticity corresponding to appropriate in vivo persistence in the peritoneal cavity played a critical role in preventing post-surgical adhesions. As for a dressing for cutaneous wound healing, Xu et al. reported a PLGA-PEG-PLGA-based thermogel dressing containing teicoplanin (TPN), a glycopeptide antibiotic, for cutaneous wound repair. they revealed that the treatment using the thermogel dressing reduced inflammation response and enhanced wound healing process (e.g., disposition of collagen, angiogenesis, and wound closure) [57].

## 5. Thermoresponsive Nanocomposite Hydrogels Composed of Laponite and Block Copolymer with PEG and PLGA Blocks

In order to support the micellar network of block copolymer with PEG and PLGA blocks and achieve thermoresponsive gelation at lower solute concentrations, the addition of laponite to the copolymer solution, or the fabrication of laponite/copolymer nanocomposites, could be an effective method. Laponite, a synthetic disk-like silicate, is a biocompatible nanoparticle with a diameter of 25 nm and thickness of 1–4 nm. Laponite has a negative face charge and a positive rim charge and forms a “house of cards” structure in water. Thus, laponite suspension becomes gel around 2 wt% [58,59,60,61]. In previous studies on laponite/copolymer nanocomposites, block copolymers with PEG and poly(propylene glycol) (PPG) blocks (PEG–PPG–PEG) and with PEG and PLGA (PLGA–PEG–PLGA and PEG–PLGA) were used. In terms of T_gel_, laponite/PEG–PPG–PEG nanocomposites exhibited higher T_gel_ around 65 °C, while laponite/PLGA–PEG–PLGA and laponite/PEG–PLGA nanocomposites exhibited T_gel_ around physiological temperature [62]. Therefore, considering the use of laponite/copolymer nanocomposites for biomedical applications, laponite/PLGA–PEG–PLGA and laponite/PEG–PLGA nanocomposites could be more appropriate. In addition, it should be noted that laponite may be the only nano-additive that can be used for the fabrication of nanocomposite hydrogels with thermoresponsive gelation. As additives other than laponite, HAp, a bioactive ceramic, has also been studied especially for bone tissue engineering. For example, the microparticles of HAp was examined as an additive in 2012 and the nanoparticles of HAp was studied for a calcium delivery system as mentioned in Section 4 [51,63]. The addition of HAp enhanced the mechanical properties of thermoresponsive hydrogels, however, the copolymer concentration used for the fabrication of hydrogels was almost the same as the concentration of the conventional hydrogels (10-30 wt%) [63]. Therefore, Materials with high water content such as laponite/PLGA–PEG–PLGA and laponite/PEG–PLGA nanocomposites with low solute concentration could be advantageous compared to the conventional hydrogels due to the high compatibility of water to cells/biomolecules. In this section, a quick review of thermoresponsive nanocomposite hydrogels composed of laponite and block copolymers with PEG and PLGA blocks is presented.

**Figure 7:**
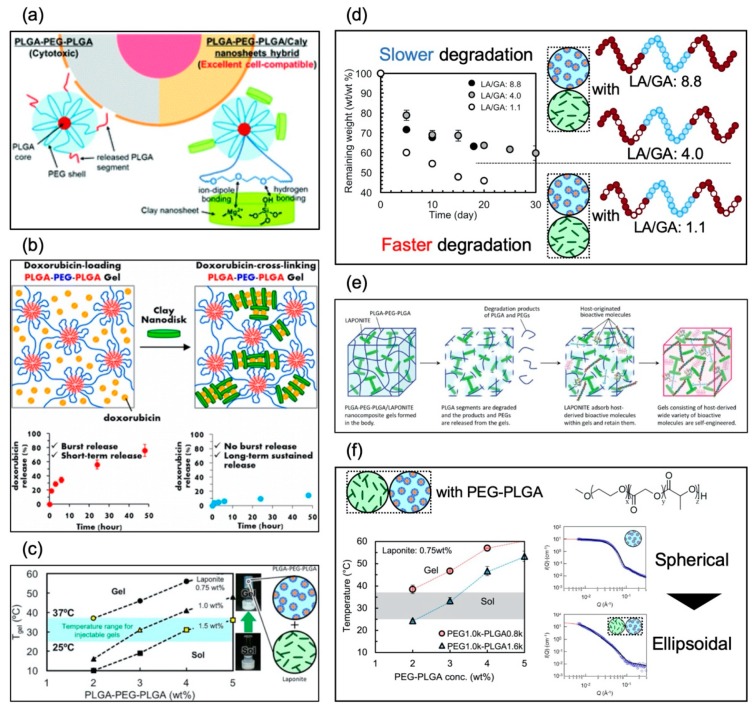
Graphical abstracts of nanocomposite systems reported in (**a**) June 2014 (reproduced from [64] with permission from the Royal Society of Chemistry), (**b**) February 2015 (reprinted with permission from [65] Biomacromolecules, 16, Koji Nagahama et al., Self-assembling polymer micelle/clay nanodisk/doxorubicin hybrid injectable gels for safe and efficient focal treatment of cancer, 880–889, Copyright (2015) American Chemical Society), (**c**) March 2017 (reprinted from [66] Polymer, 115, Makoto Miyazaki et al., PEG-based nanocomposite hydrogel: Thermoresponsive sol-gel transition controlled by PLGA–PEG–PLGA molecular weight and solute concentration, 246–254, Copyright (2017),with permission from Elsevier), (**d**) November 2017 (reprinted from [67] Polymer Degradation and Stability, 147, Kitagawa Midori et al., PEG-based nanocomposite hydrogel: Thermo-responsive sol-gel transition and degradation behavior controlled by the LA/GA ratio of PLGA–PEG–PLGA, 222–228, Copyright (2017), with permission from Elsevier), (**e**) January 2018 (reproduced from [68] with permission from the Royal Society of Chemistry), and (**f**) February 2019 (reprinted from [69]).

Table 4 shows a summary of the development history of thermoresponsive nanocomposite hydrogels. Oyama, Maeda, and Nagahama et al. first reported on a nanocomposite approach to developing biodegradable thermoresponsive hydrogels with excellent cell compatibility (Figure 7a) [64]. We revealed that thermoresponsive nanocomposite hydrogels with low CGC could be obtained by blending PLGA–PEG–PLGA triblock copolymers and laponite, and that the laponite was able to promote the thermoresponsive formation of networks for PLGA–PEG–PLGA copolymers/micelles. We also revealed excellent cell compatibility from hemolysis assay and cell culture test on and in the hydrogels. Then, in 2015, Nagahama et al. reported on a delivery system for DOX from thermoresponsive nanocomposite hydrogels (Figure 7b) [65], revealing that DOX worked as a crosslinking agent. They also revealed that long-term sustained release of DOX from thermoresponsive nanocomposite hydrogels without initial burst release could be achieved. They also revealed that a single injection of DOX-loaded thermoresponsive nanocomposite hydrogel exhibited long-term sustained antitumor activity in vivo. In 2017, Miyazaki and Maeda et al. reported on controlling the T_gel_ and viscoelasticity of thermoresponsive nanocomposite hydrogels by PLGA–PEG–PLGA molecular weight and solute concentration (Figure 7c) [66]. We synthesized PLGA–PEG–PLGA with a high PEG–PLGA ratio of 0.80 and prepared the nanocomposite solution by mixing laponite aqueous suspension and PLGA–PEG–PLGA aqueous solution. We revealed that discriminating the concentration combinations of laponite (from 0.75 wt% to 1.5 wt%) and PLGA–PEG–PLGA (from 2.0 wt% to 5.0 wt%) could effectively regulate the T_gel_ to fall between 25 °C and 37 °C (physiological temperature), and the discriminating concentration combinations simultaneously controlled the storage and loss moduli of the hydrogels. Kitagawa and Maeda et al. also reported on controlled degradation of thermoresponsive nanocomposite hydrogel composed of laponite and PLGA–PEG–PLGA (Figure 7d) [67]. We synthesized PLGA–PEG–PLGA with a high PEG/PLGA ratio of ~0.8 and with different LA/GA ratios and prepared the nanocomposite solution by mixing laponite aqueous suspension and PLGA–PEG–PLGA aqueous solution. We revealed that the T_gel_ at physiological temperature (25–37 °C) was achieved regardless of the LA/GA ratio, and the decomposition rates of thermoresponsive nanocomposite hydrogel at 37 °C could be regulated by adjusting the LA/GA ratio. In 2018, Nagahama et al. reported on ECM adsorption properties and in vitro cell compatibility of thermoresponsive nanocomposite hydrogel, and in vivo tissue reconstruction and functional recovery using the hydrogel (Figure 7e) [68]. They revealed that thermoresponsive nanocomposite hydrogel strongly adsorbed and retained ECM molecules such as collagen and heparin within gels. They also revealed that human dermal fibroblasts (HDFs) cultured on thermoresponsive nanocomposite hydrogels exhibited high cell viability and proliferation, and various kinds of human cells, such as HDFs, umbilical vein endothelial cells (HUVECs), and adipose-derived mesenchymal stem cells (ADSCs), encapsulated into thermoresponsive nanocomposite hydrogel exhibited significantly higher cell viability, proliferation, and three-dimensional organization compared to PLGA–PEG–PLGA and laponite hydrogels. They further revealed that transplantation of mouse myoblast C2C12 cells using thermoresponsive nanocomposite hydrogels dramatically enhanced tissue regeneration and functional recovery in model mice with skeletal muscle injury, whereas no recovery was observed for transplantation using PLGA–PEG–PLGA hydrogels. In 2019, Maeda et al. reported on thermoresponsive nanocomposite hydrogel composed of laponite and PEG–PLGA diblock copolymers (Figure 7f) [69]. We synthesized PEG–PLGA with a high PEG-PLGA ratio of 1.27 and prepared the nanocomposite solution by mixing laponite aqueous suspension and PEG–PLGA aqueous solution. We revealed that thermoresponsive gelation could be achieved by using PEG–PLGA, and the T_gel_ fell between 25 °C and 37 °C (physiological temperature) by controlling the PEG-PLGA and laponite concentration. We also revealed that PEG-PLGA copolymers were on the surface of the laponite and could trigger the thermoresponsive connection of the preformed laponite network.

## 6. Conclusions

In this review, the current trends of research on block copolymers composed of PEG and PLGA during the last 5 years (2014–2019) including new parameters affecting thermoresponsive gelation, structural findings from new analytical methods, research on biomedical applications including drug delivery systems and regeneration medicine, and nanocomposites composed of copolymers with PEG and PLGA blocks and nanomaterials (laponite) were comprehensively summarized and discussed. In order to utilize thermoresponsive hydrogels composed of block copolymers with PEG and PLGA blocks as biomedical devices with high efficacy, further accurate analysis and verification experiments such as computer simulations and structural analysis using new analytical methods could be consecutively conducted. In addition, new approaches including dynamic crosslinking and the nanocomposite approach are also useful in order to fabricate highly functionalized thermoresponsive hydrogels and add new functions.

## Figures and Tables

**Table 1:** Parameters affecting thermoresponsive gelation.

Parameter	Findings	Ref.
Salt concentration	Re-entrant T_gel_ decreased as salt concentration increased	[19]
Type of ion	Re-entrant T_gel_ decreased as salting-out ability increased according to Hofmeister series	[19]
MWD under similar Mw or Mn	Solubility increased as Mw/Mn increased	[20]
	CMC increased as Mw/Mn increased	
	CGC increased with increased Mw/Mn under both given Mw and given Mn	
	T_gel_ increased with increased Mw/Mn under both given Mw and given Mn	
MWD of PEG block	Gel-to-sol or sol-to-gel transition only with appropriate Mw and MWD	[21]
	Wider Mw/Mn of PEG block sometimes led to the coexistence of sol-to-gel transition upon cooling and upon heating	
PLGA/PEG ratio	Linear relation between T_gel_ and PLGA/PEG ratio	[22]
PEG molecular weight	T_gel_ dependency on PLGA/PEG ratio became less obvious as PEG molecular weight increased	[22]
Positional isomer of coupling agent	o-PC had smaller coil size compared to m-PC and p-PC	[23]
	o-PC exhibited lower T_gel_ and higher modulus compared to m-PC and p-PC	

MWD, molecular weight distribution; Mw, weight-average molecular weight; Mn, number-average molecular weight; CMC, critical micelle concentration; CGC, critical gel concentration; PEG, polyethylene glycol; PLGA, poly(lactic acid-*co*-glycolic acid); PC, phthaloyl dichloride.

**Table 2:** Methods of structural analysis of thermoresponsive hydrogels.

Method	Analytical Target	Ref.
SANS	Micellar structure and network structures composed of micelles	[24]
3D DLS	R_h_ in concentrated solution/turbid system	[25]
FRET	Nanoscale distance	[25]
DPD simulation	Morphology of copolymers in water	[26]
Monte Carlo simulation	Phases and condensed-state structures	[23,25]

SANS, small-angle neutron scattering; DPD, dissipative particle dynamics; DLS, dynamic light scattering; FRET, fluorescence resonance energy transfer.

**Table 3:** Biomedical applications of thermoresponsive hydrogels using block copolymers with PEG and PLGA blocks.

Used for	Drug/Cell	Dosage from	Ref.
Cancer treatment (osteosarcoma)	DOX	Hydrogel	[27]
Cancer treatment	Irinotecan	Hydrogel	[28]
Ophthalmic treatment	Model drug	Hydrogel	[29]
Treatment of posterior segment eye disease	Dexamethasone	Hydrogel	[30]
Treatment of glaucoma	Cyclosporine	Hydrogel	[31]
Corneal neovascularization	Metformin Levofloxacin HCl	Hydrogel	[32]
Treatment of congenital sensorineural hearing loss	Cidofovir/ganciclovir Dexamethasone	Hydrogel	[33]
Helminth emesis	Albendazole sulfoxide	Hydrogel	[34]
Treatment of opioid and alcohol addiction	Naltrexone HCl	Hydrogel	[35]
Postoperative pain relief	Ropivacaine HCl	Hydrogel	[36]
Cancer treatment (Hepatoma)	DOX (polymer conjugate) DTX	Hydrogel	[37]
Cancer treatment (Breast)	DOX	Liposome in hydrogel	[38]
Cancer treatment	Cytarabine HCl (complex with AOT)	Hydrogel	[39]
Treatment of Parkinson syndrome/cocaine dependence	Amantadine (complex with OA)	Hydrogel	[40]
Treatment of epilepsy	Oxcarbazepine	Emulsome in hydrogel	[41]
Treatment of glaucoma	Brimonidine (complex with LDH)	Hydrogel	[42]
Postoperative treatment after ocular surgery	Moxifloxacin Dexamethasone Levobunolol	MPs in hydrogel	[43]
Treatment of diabetes	Liraglutide	Hydrogel	[44]
Growth	Growth hormone–releasing peptide (GHRP-6)	Hydrogel	[45]
Treatment of diabetes	Exenatide	MPs in hydrogel	[46]
Inhibition of bone resorption	hCT (complex with CB7)	Hydrogel	[47]
Anti-osteopenia therapy	Salmon calcitonin (complex with OCA)	Hydrogel	[48]
Cancer treatment (osteosarcoma)	PLK1shRNA/PEI-Lys DOX	Hydrogel	[49]
Bone tissue regeneration	Simvastatin	Hydrogel	[50]
Bone tissue engineering	Calcium cation	HAp in hydrogel	[51]
Treatment of femoral defects	BMP-2	Hydrogel	[52]
Repair of articular cartilage defects	Stem cells (BMMSCs)	–	[53]
ESD	–	–	[54,55]
Postoperative adhesion prevention	–	–	[56]
Cutaneous wound healing	Teicoplanin	Hydrogel	[57]

ESD, endoscopic submucosal dissection; PEI-Lys, polylysine-modified polyethylenimine; DOX, doxorubicin; AOT, sodium bis(2-ethylhexyl) sulfosuccinate; DTX, docetaxel; LDH, layered double hydroxide; MP, microparticle; BMP, bone morphogenetic protein; HAp, hydroxyapatite; hCT, human calcitonin; CB7, cubit 7; OCA, oxidized calcium alginate; BMMSCs, bone marrow mesenchymal stem cells; OA, oleic acid.

**Table 4:** Development history of thermoresponsive nanocomposite hydrogels.

Date	Contents	Ref.
June 2014	Proposal of nanocomposite approach to develop thermoresponsive nanocomposite hydrogels Confirmation of excellent cell compatibility	[64]
February 2015	Confirmation of sustained release of DOX from thermoresponsive nanocomposite hydrogels Confirmation of antitumor efficacy of DOX-loaded thermoresponsive nanocomposite hydrogels	[65]
March 2017	Controlling T_gel_ by PLGA–PEG–PLGA molecular weight and solute concentration Structural analysis of thermoresponsive nanocomposite hydrogels by Cryo-TEM and small-angle X-ray scattering (SAXS)	[66]
November 2017	Controlling degradation behavior by LA/GA ratio of PLGA–PEG–PLGA	[67]
January 2018	Confirmation of ECM adsorption within thermoresponsive nanocomposite hydrogels Confirmation of cell viability and proliferation using human cells Confirmation of enhance tissue regeneration and functional recovery though cell transplantation in model mice	[68]
February 2019	Utilizing water-soluble PEG–PLGA diblock copolymers to obtain thermoresponsive nanocomposite hydrogels Structural analysis of thermoresponsive nanocomposite hydrogels by SANS	[69]
